# Pharmacologic inhibition of reactive gliosis blocks TNF-*α*-mediated neuronal apoptosis

**DOI:** 10.1038/cddis.2016.277

**Published:** 2016-09-29

**Authors:** Izhar Livne-Bar, Susy Lam, Darren Chan, Xiaoxin Guo, Idil Askar, Adrian Nahirnyj, John G Flanagan, Jeremy M Sivak

**Affiliations:** 1 Department of Vision Sciences, Krembil Research Institute, University Health Network, Toronto, Ontario, Canada; 2 School of Optometry, University of California at Berkeley, Berkeley, CA, USA; 3 Department of Ophthalmology and Vision Science, University of Toronto, Toronto, Ontario, Canada

## Abstract

Reactive gliosis is an early pathological feature common to most neurodegenerative diseases, yet its regulation and impact remain poorly understood. Normally astrocytes maintain a critical homeostatic balance. After stress or injury they undergo rapid parainflammatory activation, characterized by hypertrophy, and increased polymerization of type III intermediate filaments (IFs), particularly glial fibrillary acidic protein and vimentin. However, the consequences of IF dynamics in the adult CNS remains unclear, and no pharmacologic tools have been available to target this mechanism *in vivo*. The mammalian retina is an accessible model to study the regulation of astrocyte stress responses, and their influence on retinal neuronal homeostasis. In particular, our work and others have implicated p38 mitogen-activated protein kinase (MAPK) signaling as a key regulator of glutamate recycling, antioxidant activity and cytokine secretion by astrocytes and related Müller glia, with potent influences on neighboring neurons. Here we report experiments with the small molecule inhibitor, withaferin A (WFA), to specifically block type III IF dynamics *in vivo.* WFA was administered in a model of metabolic retinal injury induced by kainic acid, and in combination with a recent model of debridement-induced astrocyte reactivity. We show that WFA specifically targets IFs and reduces astrocyte and Müller glial reactivity *in vivo*. Inhibition of glial IF polymerization blocked p38 MAPK-dependent secretion of TNF-*α*, resulting in markedly reduced neuronal apoptosis. To our knowledge this is the first study to demonstrate that pharmacologic inhibition of IF dynamics in reactive glia protects neurons *in vivo*.

Astrocyte reactivity (reactive gliosis) is an early pathological feature common to most neurodegenerative diseases, yet its regulation and impact remains poorly understood. In the healthy central nervous system (CNS), astrocytes coordinate homeostatic vascular perfusion, free radical detoxification and neurotransmitter recycling.^1, 2^ Injury or stress induces a phenotypic switch, whose cardinal features are cellular hypertrophy and increased expression and polymerization of type III intermediate filaments (IFs), particularly glial fibrillary acidic protein (GFAP).^3, 4, 5^ The role of intermediate filaments in reactive gliosis remains unclear.^3, 6, 7, 8, 9^ Genetic deletion of IFs GFAP and vimentin have been shown to promote axonal outgrowth and regeneration in developing neurons and models of CNS injury,^10, 11, 12^ yet result in developmental defects to inner retinal function^13^ and increased damage in models of Alzheimer's disease.^14^ Genetically, GFAP gain of function mutations associated with Alexander's disease induce a p38 mitogen-activated protein kinase (MAPK)-dependent pathology.^15^ However, no pharmacologic tools have been available to specifically modulate and explore this reactive switch in the context of pathological CNS injury. Consequently, strategies to therapeutically target the reactive switch have remain challenging to explore.

Withaferin A (WFA) is a small molecule withanolide that is a potent and specific inhibitor of type III intermediate filament dynamics.^16, 17, 18^ Its activity has been most closely studied with respect to vimentin rearrangement and phosphorylation in the context of angiogenesis, fibrosis and cancer, through downstream effects on inflammatory signaling and cell proliferation.^19, 20, 21, 22, 23, 24^ Interestingly, WFA has been reported to regulate vimentin-mediated activation of MAPKs in a context dependent manner, as well as NF*κ*B.^25, 26^ Recently Bargagna-Mohan *et al.*^27^ reported that, in addition to vimentin, WFA also binds covalently to GFAP at cysteine 294. In these studies WFA impaired GFAP filament assembly and polymerization in cultured astrocytes, and *in vivo* in retinal astrocytes and related Müller glia in a model of injury-induced gliosis.^27^ Therefore, WFA presents a novel tool to test the pharmacologic blockade of intermediate filament remodeling during gliosis. However, the consequences of WFA disruption of IFs on neuronal damage has not been studied.

We have previously used the retina as a uniquely accessible model to study the regulation of astrocyte stress responses, and their influence on retinal neuronal survival.^28, 29, 30^ In the human and rodent eye retinal ganglion cells (RGCs) and amacrine cells of the inner retina maintain a delicate homeostatic balance and are particularly vulnerable to excitotoxic and metabolic damage, mediated in part through non-cell autonomous interactions with neighboring glia.^31, 32, 33, 34^ In addition, our work and others has implicated signaling through p38 MAPKs as key regulators of glutamate recycling, antioxidant activity, and cytokine secretion in neighboring stress-activated retinal astrocytes and Müller glia.^29, 35, 36, 37^ Here we take advantage of a model of induced retinal astrocyte reactivity to establish whether WFA, and the selective p38 MAPK inhibitor SB203580 (SB), affect neuronal apoptosis in a mouse model of excitotoxic injury.

## Results

### WFA and SB treatment blocks glial reactivity and rescues retinal neurons from apoptosis

**Figure 1:**
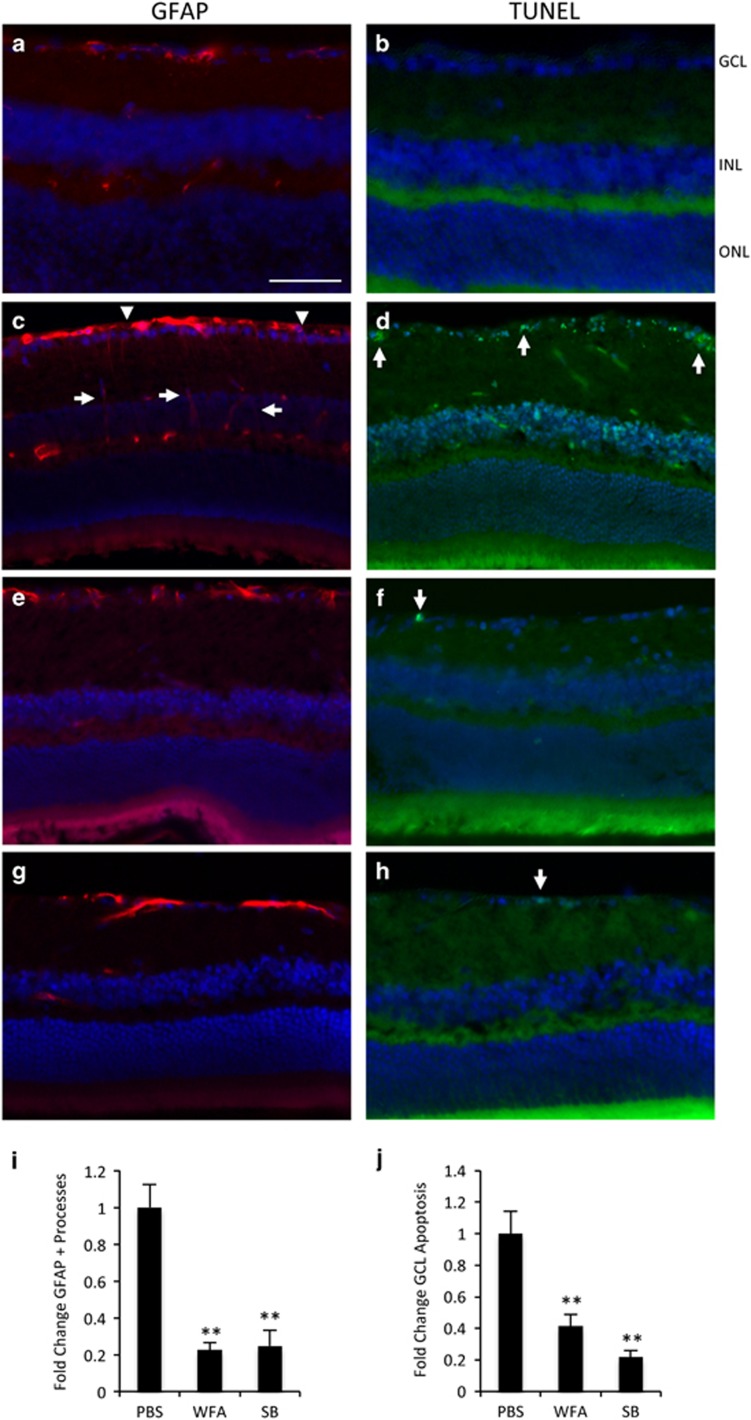
WFA and SB inhibit retinal gliosis and apoptosis. (**a** and **b**) Intraocular injection of vehicle alone leads to no increase in GFAP stained glial fibers or TUNEL stained apoptotic cells. (**c** and **d**) Injection of KA induces increased GFAP staining of retinal astrocytes (arrowheads), and induced staining in Müller fibers (arrows) and apoptotic death of cells in the GCL (arrows) and INL by 18 h. (**e** and **f**) Treatment with WFA blocks the increased GFAP and TUNEL staining. (**g** and **h**) Treatment with SB203580 (SB) similarly blocks the increased GFAP and TUNEL staining. (**i**) Quantification of the GFAP and TUNEL results from multiple animals showing fold change compared with contralateral control eyes (bars indicate S.E., **P<0.01, *n*⩾4). Scale bar indicates 50 *μ*m. GCL, ganglion cell layer; INL, inner nuclear layer; ONL, outer nuclear layer. All of the following panels and images are oriented in the same way

For the present series of experiments we utilized a well-established excitotoxic model of inner retinal injury that is closely associated with reactivity of resident astrocytes and Müller glia.^33, 38, 39, 40, 41, 42^ Intraocular injection of kainic acid (KA) consistently generated rapid accumulation of GFAP in retinal astrocytes and Müller fibers (Figure 1a and c), and apoptosis of RGCs and amacrine cells in the ganglion cell layer (GCL) and inner nuclear layer (INL) by 18 h (Figure 1b and d). This method facilitates rapid and accurate quantification of markers for the resulting neuronal apoptosis and glial reactivity.^28^ In order to study the influence of IF dynamics on this model, we first tested the efficacy of WFA and the selective p38*α* and *β*-MAPK inhibitor SB203580 (SB).

Mice were administered either WFA (2 mg/kg), or vehicle control. WFA strongly reduced GFAP levels in inner retinal astrocytes and Müller fibers following KA challenge (Figure 1e). WFA treatment also resulted in a corresponding marked reduction in inner retinal apoptosis in the GCL and INL compared with control mice (Figure 1f). As we have previously determined that blocking p38 MAPK reduces activation of retinal astrocytes *in vitro*,^29^ we also tested intravitreal administration of SB (2 mM), which similarly strongly reduced GFAP staining (Figure 1g), and markedly decreased neuronal apoptosis compared with vehicle (Figure 1h).

To quantify the extent of retinal gliosis and neuronal apoptosis in these studies, we followed established procedures to count activated Müller glia and GCL death. However, similar patterns were also observed in the INL (Figure 1a–h). For gliosis quantification, the number of GFAP-positive processes crossing the inner plexiform layer were counted across the retina in multiple sections through the level of the optic nerve head, and averaged for multiple animals.^43^ On adjacent sections TUNEL-positive cells in the GCL were similarly quantified across the retina and averaged by established methods.^28, 44, 45, 46^ These analyses confirmed a highly significant 80% reduction in GFAP-positive processes for both WFA and SB, and 60% and 80% reductions in GCL apoptosis, respectively (Figure 1i and j). Therefore, administration of WFA or SB effectively blocked KA-induced reactivity and apoptosis in the retina.

### WFA treatment compromises intermediate filament polymerization

**Figure 2:**
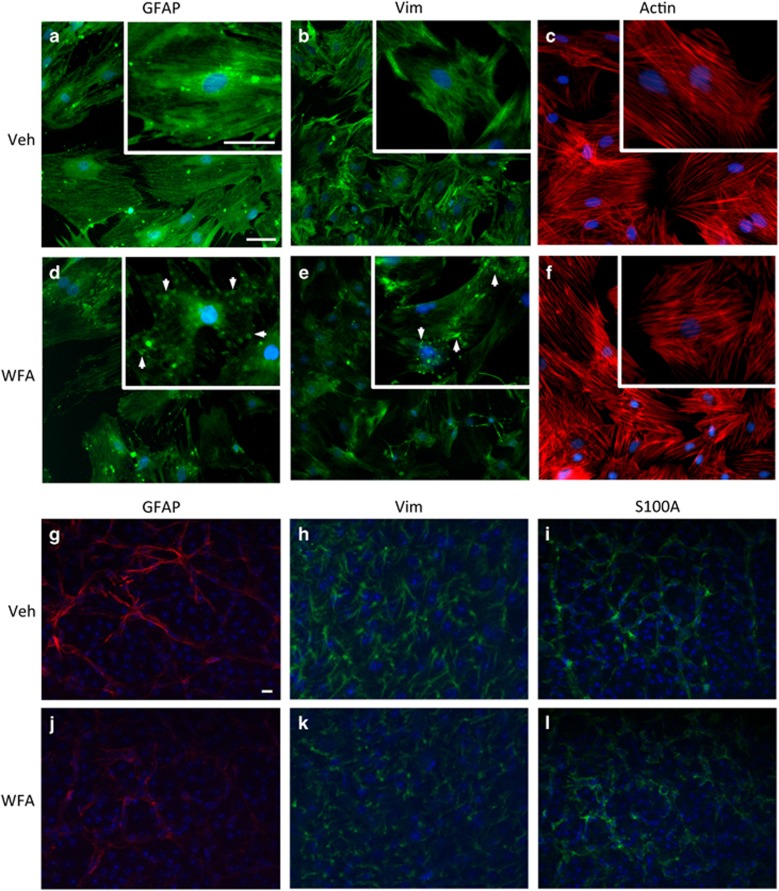
WFA treatment inhibits IF dynamics. (**a**–**f**) Treatment of cultured retinal astrocytes with 2.0 *μ*m WFA resulted in reduced staining for GFAP (**a** and **d**), and vimentin (vim; **b** and **e**), compared with control treated cells after 8 h. At higher magnifications (insets), filamentous staining for GFAP and vimentin was disrupted, resulting in formation of IF aggregates (arrows). In comparison, filamentous actin remained unaffected (**c** and **f**). (*n*=3, scale bars indicate 100 *μ*m. Identical exposures were used for all treatments). (**g**–**l**) WFA treatment *in vivo* similarly disrupts GFAP and vimentin in retinal flatmounts, but not S100A. (*n*=3, scale bars indicate 100 *μ*m. Identical exposures were used for all treatments)

In order to confirm the direct activity of WFA on astrocyte IFs, we treated cultured retinal astrocytes with the drug for subsequent analyses by fluorescent microscopy. Highly enriched retinal astrocyte cultures were established from adult rats according to our published methods.^28, 29, 30^ We have previously shown that these cells present typical astrocytic morphology, and a variety of specific markers, including GFAP, vimentin, Pax-2, glutamine synthetase (GS) and S100A.^29^ They also robustly respond to metabolic and oxidative stress with p38 MAPK-dependent changes in activation markers, secreted cytokines and antioxidants.^29^ In the present study, vimentin and GFAP filaments were assessed in retinal astrocytes by fluorescent microscopy following treatment with 2** ***μ*M WFA or vehicle. Both vimentin and GFAP showed reduced filamentous signal and the formation of short aggregates following WFA exposure (Figure 2a–b and d–e). In comparison, staining for actin showed no effect on filament assembly (Figure 2c and f). These specific WFA effects on IF polymerization are consistent with previous reports.^16, 27^

To confirm these effects *in vivo*, retinas from control and WFA-treated mice were also flatmounted and probed for GFAP and vimentin, followed by imaging with confocal microscopy. The intensity and extent of GFAP and vimentin filaments was reduced in WFA-treated animals compared with vehicle, consistent with our *in vitro* results (Figure 2g–h and j–k). By comparison, staining for the astrocyte marker S100 was not strongly affected and indicated similar numbers of cells in treated and vehicle samples, but with apparent changes to astrocyte morphology (Figure 2i and l). Likewise, there were no WFA-induced changes in staining for GS in retinal sections (Supplementary Figure 1). In general the treated astrocytes appeared smaller with fewer processes (Figure 2j–l).

### A non-invasive injury model of induced retinal glial activation

Demonstrating IF-dependent effects *in vivo* is challenging due to developmental and compensatory effects of induced gene deletions, and the intimate associations between glial reactivity and neurodegeneration. For this purpose we took advantage of a recently described model of induced retinal glial activation. Bargagna-Mohan *et al.* reported that mechanical corneal debridement-induced sensitive and robust retinal astrogliosis, but did not cause any apparent changes in retinal morphology or pathology compared with untreated eyes.^27, 47^ This new model of retinal astrocyte reactivity provides a unique opportunity for investigating the influence of injury-induced glial reactivity on neurovascular tissue. We decided to use this assay to test the reactivity-dependent effects of WFA and SB. In order to adapt the model for this purpose, we first set out to reproduce and expand the previously reported results.

**Figure 3:**
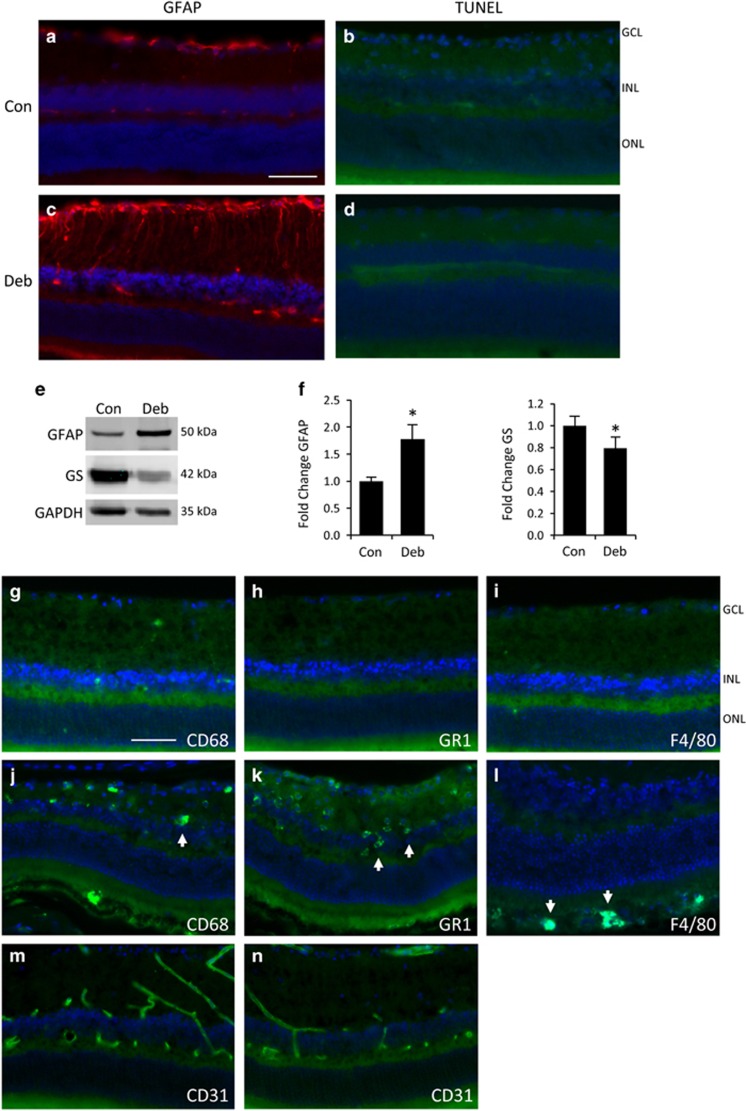
Rapid retinal glial reactivity is induced by corneal injury in the absence of cell death or inflammation. (**a** and **b**) Non-debrided control eyes show no evidence of gliosis or cell death. (**c** and **d**) Mechanical debridement of the corneal epithelium in the contralateral eye results in strongly increased GFAP staining in retinal astrocytes and Müller glia by 7 days (**c**), but no evidence of cell death or disrupted morphology (**d**). (**e**) Western blots from whole retina lysates show increased GFAP and decreased GS. (**f**) Densitometry and statistical analyses from multiple blots confirming the changes in **e** (bars represent SE, *P<0.05, *n*=3 animals). (**g**–**i**) Staining of debrided retinas for activated microglia, neutrophils and macrophages, was largely negative with CD68, GR-1 and F4/80 antibodies, respectively. (**j**–**l**) Corresponding antibody-positive controls from eyes treated with 150 mM NaOH. (**m** and **n**) There was no change in retinal blood vessel staining with the endothelial marker CD31 between control (**m**) or debrided eyes (**n**). (Scale bar indicates 50 *μ*m, Con; control, Deb; debrided.)

According to well-established methods the corneal epithelium was transiently removed by gentle mechanical debridement, according to our published procedure.^48^ The epithelium resurfaces within 1–2 days accompanied by transient corneal inflammation and neovascularization. Bargagna-Mohan *et al.* reported that by 7 days post injury a robust increase in GFAP is induced in astrocytes and Müller fibers.^27, 47^ Following the same procedure we also observed a strong increase in retinal GFAP staining compared with non-debrided controls (Figure 3a and c). Also consistent with the previous reports, there was no major change to retinal morphology or evidence of apoptosis (Figure 3b and d). As a second confirmation of increased gliosis, we performed western blotting for GFAP, and also for GS; a key glutamate recycling enzyme that is characteristically reduced in activated astrocytes.^49, 50^ Consistent with the immunofluorescence images, GFAP was significantly increased and GS significantly decreased in retinas isolated from debrided eyes, compared with non-debrided controls (Figure 3e and f).

To control for possible inflammatory effects we probed retinas from debrided eyes for CD68, GR-1 and F4/80, for evidence of activated microglia, neutrophils and macrophages, respectively (Figure 3g–i). Little or no staining was found, compared with positive controls (Figure 3j–l). We also stained retinas from debrided eyes for vascular changes with CD31 and observed no differences from non-debrided controls, as previously reported^27^ (Figure 3m and n). Therefore, we confirmed that this model does induce robust and consistent astrocyte and Müller glial reactivity in the inner retina with no evidence of accompanying damage or inflammatory response.

### Activation of retinal astrocytes increases neuronal vulnerability in an IF and p38 MAPK-dependent manner

Our initial experiment treatment with WFA or SB potently blocked KA-induced astrocyte activation and neuronal apoptosis, but each inhibitor could also act through additional mechanisms to directly affect neurons. In particular, SB has been proposed to directly inhibit p38-dependent RGC apoptosis.^51^ Therefore, we designed a combined experiment in which KA challenge was combined with, or without, induced astrocyte activation in order to isolate the influence of the IF-mediated reactivity response.

**Figure 4:**
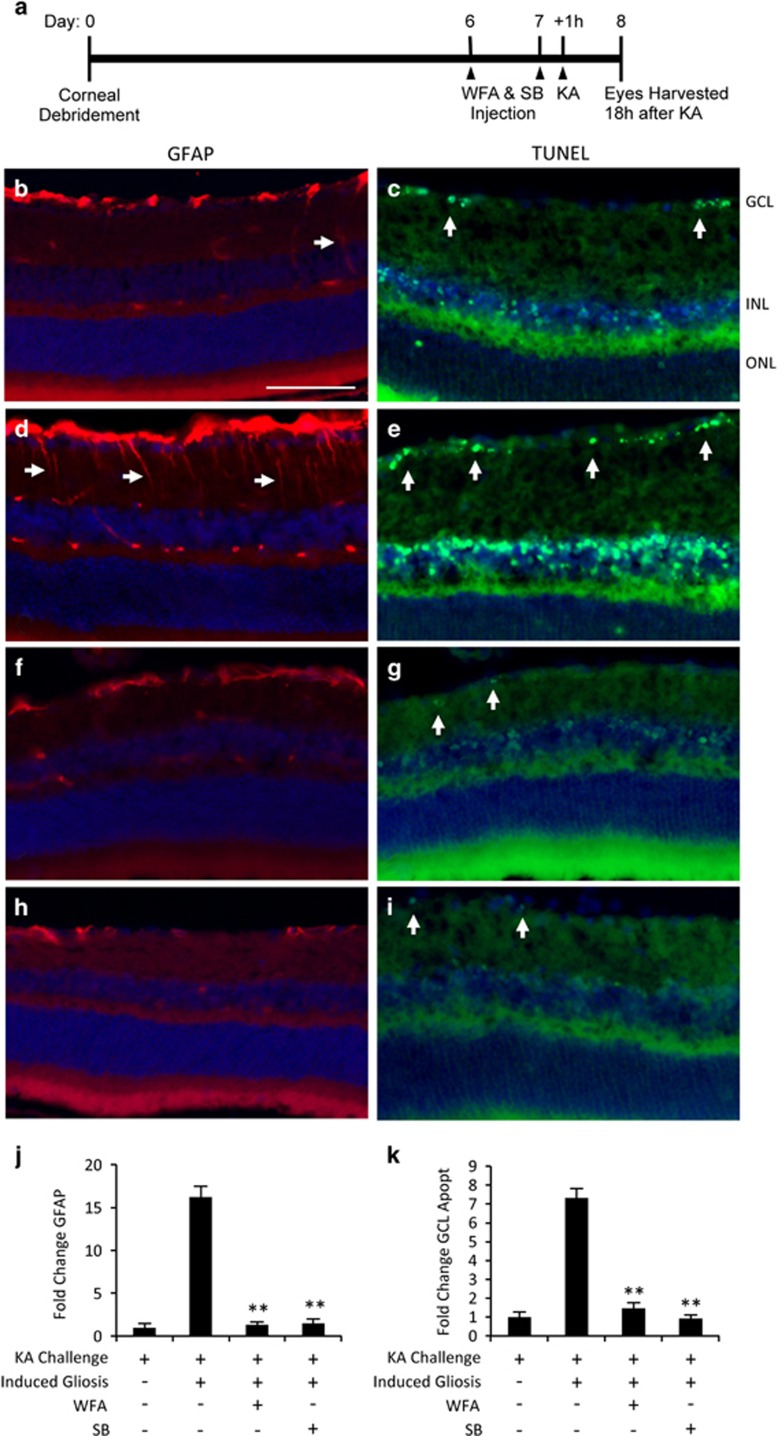
Induced glial reactivity promotes inner retinal apoptosis and is rescued by IF and p38 MAPK inhibition. (**a**) Schematic for induction of retinal gliosis by corneal debridement, followed by drug or vehicle treatments and KA challenge. (**b** and **c**) KA challenge induced rapid gliosis and GCL death as previously. (**d** and **e**) Prior corneal debridement-induced increased GFAP accumulation and increased GCL apoptosis following KA insult (arrows). Treatment with WFA (**f** and **g**) or SB (**h** and **i**) rescued the debridement-induced gliosis and cell death. (**j** and **k**) Quantification of the results showing that gliosis induced GCL death in WFA and SB-treated animals is significantly reduced (bars are S.E., **P<0.01, *n*⩾6 animals). Scale bar indicates 50 *μ*m

In this combined design retinal astrogliosis was induced by debridement injury one week before KA challenge, along with each drug treatment or vehicle (Figure 4a). In naive, non-debrided eyes, KA treatment-induced moderate glial activation and GCL apoptosis as previously described (Figure 4b and c). In comparison, corneal debridement-induced massive reactivity in combination with KA challenge (Figure 4d). This combination also produced a marked increase in GCL apoptosis compared with KA alone (Figure 4e). Quantification of these results demonstrated a highly significant 16-fold increase in glial reactivity in debrided eyes compared with KA alone, and a 7-fold increase in apoptosis (Figure 4j and k). However, these increases were completely rescued by administration of either WFA or SB (Figure 4f–k). This experiment therefore provides evidence that induced glial reactivity increases the vulnerability of retinal neurons to excitotoxic injury, and that WFA and SB mediate their protective effects through blocking this switch.

### WFA inhibits p38 MAPK-dependent TNF-*α* secretion

**Figure 5:**
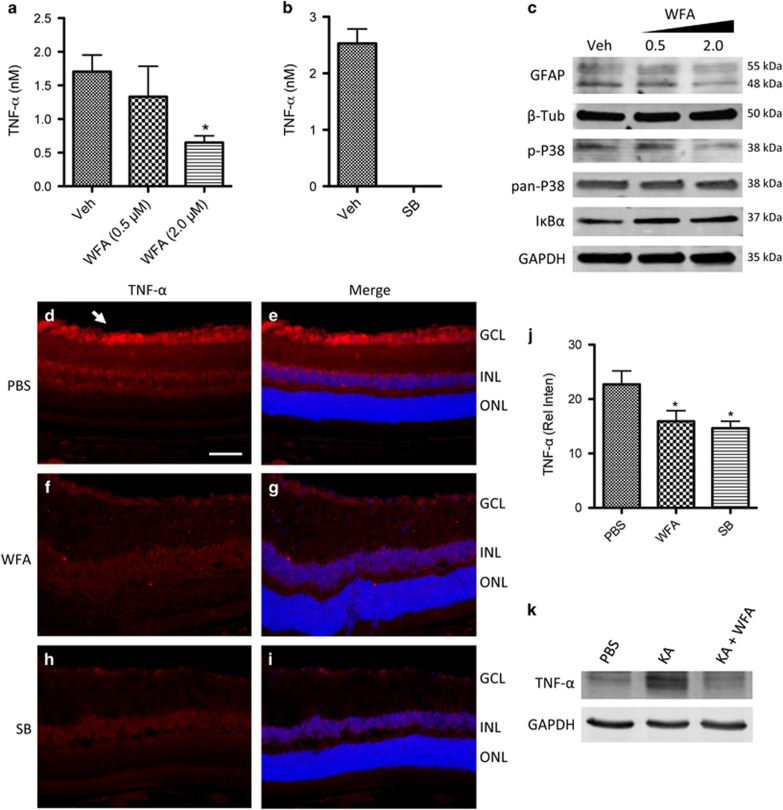
WFA treatment inhibits p38 MAPK-dependent TNF-*α* secretion in the inner retina. (**a**) Conditioned media from astrocytes treated with WFA showed a dose-dependent decrease in TNF-*α* concentrations (*P<0.05, *n*=3 cultures). (**b**) Conditioned media from astrocytes treated with 15 *μ*m SB showed a complete loss of detectable TNF-*α* (*n*=3 cultures). (**c**) Western blot showing increasing concentrations of WFA resulted in reduced GFAP protein and phospho-p38 MAPK (p-p38), compared with pan-p38 MAPK. In comparison there was no change in *β*-tub, I*κ*B*α*, or a GAPDH loading control. (**d** and **e**) Staining for TNF-*α* (red) appears strongly in the inner retina in KA challenged eyes (arrow). (**f** and **g**) Treatment with WFA, or (**h** and **i**) SB, reduced TNF-*α* staining. Cell nuclei are indicated by Dapi staining in all images (blue). (**j**) Quantification of TNF-*α* intensity across the GCL confirms a reduced signal in WFA- or SB-treated eyes following KA challenge (*P<0.05, *n*=3 animals per group). Scale bar represents 50 *μ*m. (**k**) Western blot of retinal lysates similarly shows increased TNF-*α* signal following KA challenge that is blocked by WFA treatment, compared with GAPDH loading control (*n*=4 animals per lane)

Neuronal apoptosis in the inner retina following disease and excitotoxic injury has been previously shown to be dependent on cytokine signals secreted by adjacent astrocytes.^33, 34, 50, 52^ Therefore we investigated WFA- and SB-regulated cytokines that might explain the IF-dependent effects in our models. Starting with cultured astrocytes, conditioned media was collected from treated cells and subjected to multiplex cytokine analyses. In this case the cells were stressed by serum deprivation to facilitate collection and analyses of conditioned media. Surprisingly, out of a panel of 27 cytokines and growth factors assessed, only TNF-*α* levels were significantly altered by WFA, although, negative trends were also identified for IL-1*α* and *β*, and EGF, and a positive trend for IL-10 (Table 1). TNF-*α* concentrations were strongly reduced by WFA treatment, in a dose-dependent manner to 2 *μ*M (Figure 5a). To test whether this TNF-*α* signal was also dependent on downstream p38 MAPK signaling, cells were alternatively treated with SB, which we have previously reported to effectively block reactivity markers.^29^ SB treatment reduced TNF-*α* levels to below detection (Figure 5b). Western blotting demonstrated that increasing concentrations of WFA reduced GFAP in cultured retinal astrocytes, but not the intermediate filament *β*-tubulin (*β-*tub) (Figure 5c). Furthermore, phospho-p38 MAPK signal (p-p38) was reduced compared with total p38 (Figure 5c), consistent with previous reports.^25, 26^ In comparison, levels of I*κ*B*α* were unaffected up to 2 *μ*M (Figure 5c). Therefore, the WFA-induced effect on TNF-*α* may be mediated through p38 MAPK in retinal astrocytes.

Glial derived TNF-*α* has been closely linked to the induction of neuronal apoptosis in a variety of acute and degenerative models, including following excitotoxic injury,^33, 53, 54, 55^ and during progression of glaucoma.^34, 56, 57^ To confirm the effects of WFA on secretion of TNF-*α*, we probed retinal sections from KA challenged and control eyes with antibodies to TNF-*α* to observe the effect of IF inhibition *in vivo*.

In KA challenged eyes TNF-*α* staining was prominently localized in the inner retina, matching the observed pattern of apoptosis in the GCL and INL, but was not present in the outer retinal layers (Figure 5d and e; Supplementary Figure 2). Correspondingly, TNF receptor 1 (TNFR1) staining primarily localized to GCL neurons (Supplementary Figure 2), consistent with previous reports.^33, 34^ In comparison, sections from KA challenged eyes treated with WFA or SB showed strongly reduced TNF-*α* signal (Figure 5f–i). This TNF-*α* signal was quantified by measuring the average staining intensity across the GCL from multiple sections per animal, for multiple animals in each treatment group. Results indicated a significant reduction of TNF-*α* in WFA- and SB-treated animals compared with control (Figure 5j). Reduced TNF-*α* signal in retinal lysates was further confirmed by western blot (Figure 5k). Thus, treatment with WFA or SB reduced TNF-*α* signal in the inner retina following injury *in vivo*.

## Discussion

Astrocyte reactivity is associated with many neurodegenerative and neurotoxic processes, including Alzheimer's disease, Parkinson's disease, stroke, diabetic retinopathy and glaucoma.^3, 32, 58, 59, 60^ This parainflammatory switch contributes to progression of non-cell autonomous disease mechanisms.^4, 61^ However, the pathogenic cascades underlying reactivity have proven difficult to study *in vivo* due to a lack of pharmacologic tools to modulate this process. Genetic deletion of vimentin or GFAP results in compensatory changes in remaining IF's. Although double knockouts of GFAP and vimentin promote axonal outgrowth and regeneration in developing neurons and after CNS injury,^10, 11, 12^ and use of glial toxins is similarly protective.^42^ Yet, these deletions also result in developmental and pathologic defects in CNS patterning and function that can alter baseline tissue homeostasis.^13, 14, 15^ In order to fully test the role of IF dynamics during glial reactivity and disease, pharmacological tools are necessary to block increased activity on a normal background. Here we have presented data demonstrating that pharmacologic inhibition of glial IF dynamics blocks p38 MAPK-dependent secretion of TNF-*α*, and dramatically reduces apoptosis in a non-cell autonomous model of excitotoxic neuronal death.

WFA is the cardinal member of a family of bioactive steroidal lactones termed ‘withanolides', derived from the *Withania somnifera* plant.^62^ It is a potent inhibitor of vimentin polymerization, and has been primarily investigated for anti-angiogenic, cytostatic, and anti-inflammatory effects, particularly through modulation of NF*κ*B and MAPK signaling. Recent studies have clarified the mechanism of WFA to specifically target type III intermediate filaments, including vimentin and GFAP.^16, 27^ Additionally, multiple labs have demonstrated that its downstream kinase and transcriptional effects, as well cytostatic effect at high concentration, are dependent on IF blocking activity.^16, 20, 25, 27, 63, 64^ Therefore, WFA provides a potent tool for probing the efficacy of IF disruption in the context of neuronal injury. Interestingly, Swarup *et al.*^65^ reported improved CNS outcomes following WFA administration in a model of amyotrophic lateral sclerosis, but did not explore these results in the context of IF inhibition.

In our experiments pharmacologic blockade of IF dynamics in reactive glia with WFA-protected retinal neurons from excitotoxic metabolic stress. In the inner retina astrocyte processes and Müller glia endfeet interact closely with RGC bodies and axons to regulate inner retinal homeostasis.^8, 31, 58^ Reactive gliosis is rapidly induced following insult to the inner retina,^7, 66^ and is associated with cytokine secretion, remodeling of the optic nerve head, loss of glutamate buffering and increased production of detoxifying enzymes.^8, 58, 67, 68, 69^ Our results suggest that the anti-apoptotic activity of WFA is mediated by inhibiting a p38 MAPK-dependent production of TNF-*α* by reactive astrocytes and Müller glia. Inhibition of IFs or p38 MAPK-reduced glial reactivity, and inhibited neuronal cell death. In contrast, a new model of induced glial reactivity markedly increased susceptibility to excitotoxic death. WFA or SB might act through additional mechanisms to directly affect neurons, as previously reported for SB.^51^ Therefore, we performed a combined experiment of KA challenge with induced glial activation to demonstrate a rescue of the reactivity-mediated injury by treatment with WFA or SB.

To explain these results we show that IF inhibition blocked p38 MAPK phosphorylation, and p38-dependent secretion of TNF-*α*. In comparison, I*κ*B*α* levels were unchanged, suggesting that NF*κ*B signaling was not strongly affected. Increased p38 phosphorylation and signaling has been closely associated with astrocyte and microglia reactivity in the context of oxidative and metabolic stress through regulation of antioxidant defense, mitochondrial function and cytokine secretion, particularly TNF-*α*.^29, 70, 71^ In addition, gain of function accumulations of mutant GFAP protein causes Alexander's disease via a p38 MAPK-dependent pathology.^15^ Accordingly, inhibition of p38 signaling is protective in models of metabolic and oxidative neuronal injury.^51, 72, 73^

A cytokine screen identified that WFA significantly reduced TNF-*α* release, although a trend for negative regulation was also identified for IL-1*α* and *β*, and EGF, and a positive trend for IL-10. IL-10 has been reported to play a role in astrocyte mediated neuroprotection, through direct and indirect mechanisms.^74, 75, 76, 77^ Therefore, these additional factors may also contribute to the observed efficacy. TNF-*α* is a key proinflammatory cytokine that binds to two major death receptors (TNFR1 and TNFR2) to induce the extrinsic apoptosis cascade.^55^ Non-cell autonomous neuronal cell death due to glial derived TNF-*α* signaling has been implicated in a diverse range of neurodegenerative and neurotoxic conditions,^55^ including excitotoxicity.^33, 53^ In the eye, TNF-*α* secretion by reactive astrocytes and Müller glia has been well established to induce RGC apoptosis in experimental models,^52, 54^ and during degenerative disease.^34, 56^ Subsequently, pharmacologic inhibition or genetic deletion of TNF-*α* signaling is protective to RGCs.^33, 54^ The present results suggest that type III IF dynamics initiate a p38—TNF-*α* signaling cascade in reactive glia that exacerbates inner retinal injury. In addition, the inhibition of p38 MAPK conversely decreased IF accumulation. As both p38 and TNF-*α* have roles in the induction of astrocyte reactivity,^29, 71, 78^ it seems likely that an autocrine loop is at play that may include additional mediators, such as IL-10. Future experiments will be required to fully elucidate this signaling cascade.

Excitotoxic damage due to elevated glutamate has been associated with many acute and chronic neurodegenerative diseases of the retina and brain, including stroke, Alzheimer's disease and glaucoma.^31, 32, 79^ The inner retina is particularly vulnerable to excitotoxic and metabolic stress, which is exacerbated through the release of TNF-*α* by neighboring astrocytes and Müller glia.^28, 33^ However, the astrocyte reactivity cascade is a complex process that has both positive and negative aspects. For example, ischemic preconditioning has been reported to have protective effects that are partially attributed to activated glia.^80, 81, 82^ The mechanism underlying this protection remains unclear, and is likely influenced by signaling through additional metabolic response pathways that may differ from the p38 driven mechanism we have described here. A key goal for the present work is the targeted inhibition of IFs, rather than complete ablation of the reactive cascade. Here we have demonstrated that pharmacologic targeting of IF dynamics modulates cytokine release and dramatically reduces neuronal cell death following acute retinal injury. It will be important for future studies to expand these findings by using WFA as a probe to further investigate the roles and mechanisms of glial IF dynamics in other neurotoxic and neurodegenerative processes.

## Materials and Methods

### Retinal injury model

All animal experiments were approved by the UHN Animal Care Committee in accordance with applicable regulations. Male C57BL/6 mice were anesthetized by i.p. injection of ketamine/xylazine. Intravitreal injections with 10 mM kainic acid (KA) were performed as previously described.^28^ Briefly, a 30 g needle was inserted tangentially into the vitreous and replaced with a Hamilton syringe to inject a volume of 2 *μ*l, followed by application of ophthalmic antibiotic ointment (BNP, Vetoquinol). For drug experiments, mice received two injections of either vehicle, 2 mg/kg WFA i.p., or 2 mM SB203580 (Selleckchem, Burlington, ON, Canada) intravitreally, the day before, and 1 h before KA challenge. Eighteen hours after KA challenge mice were euthanized by CO_2_ asphyxiation and eyes processed for histopathology. In all experiments *n* refers to the number of animals tested.

### Induced glial reactivity model

Corneal epithelial debridement was performed to induce retinal glial reactivity as previously described.^27, 48^ Briefly, anesthetized mice received topical proparacaine to the eyes (Bausch and Lomb, Vaughan, ON, Canada). Eyes were proptosed with forceps, and the corneal epithelium was gently removed with a sterile disposable scalpel, followed by application of antibiotic ointment. For some experiments, at 6 and 7 days post debridement, mice received injection of either vehicle, 2 mg/kg WFA i.p., or 2 mM SB203580 intravitreally, followed by KA challenge, as described above. The retinas were processed on day eight as described above. In all experiments *n* refers to the number of animals tested.

### Astrocyte cultures

Primary mature retinal astrocytes were isolated and cultured as previously described.^30^ Briefly, retinas were dissected out of adult Wistar rat eyes and placed in ice-cold MEM-H17 culture medium. Retinas were digested by shaking in MEM-H17 containing trypsin and DNAse, followed by trituration to disperse cell aggregates. When cultures reached confluence, the cells were placed on a rotating shaker for 6–8 h and re-plated. A glial-specific expression profile was confirmed by probing the resulting cells with a marker panel. For staining and biochemistry experiments media was replaced with serum-free media and cultured for an additional 8 h with treatment of 0.5 or 2.0 *μ*M WFA, 15 *μ*M SB or the equivalent DMSO vehicle as a control. Conditioned media was collected after 8 h and immediately frozen for multiplex cytokine analyses.

### Western blotting and cytokine profiling

Cells were lysed in RIPA buffer containing Complete Mini EDTA-free protease inhibitor (Roche) and PhosSTOP phosphatase inhibitor (Roche, Mississauga, ON, Canada). Total protein was quantified and equal concentrations were submitted to SDS-PAGE by standard methods. Proteins were transferred to PVDF membrane and probed with antibodies raised against GFAP (Sigma, St. Louis, MO, USA), GS (Abcam, Cambridge, MA, USA), phospho-p38 and pan-p38 MAPK (Cell Signaling, Danvers, MA, USA), I*κ*B*α* (Santa Cruz, Dallas, TX, USA), TNF-*α* (R&D Systems, Minneapolis, MN, USA), and GAPDH (Calbiochem, San Diego, CA, USA), and detected with appropriate IRDye secondary antibody (Li-Cor Biosciences, Lincoln, NE, USA). Blots were imaged with an Odyssey infrared imaging system (Li-Cor Biosciences). For TNF-*α* and multiplex cytokine profiling, conditioned media was snap frozen and submitted for laser bead analyses on a Bioplex 200 to detect sensitive and quantitative target protein concentrations against a standard curve (Eve Technologies).

### Immunofluorescence microscopy

Enucleated eyes were fixed in 4% PFA overnight. Eyes were then equilibrated in 30% sucrose for 12 h, embedded in OCT, and cryosectioned at 16 *μ*M. Sections were blocked and probed with primary antibodies to GFAP (Sigma), GS (Sigma), CD68 (Biolegend, San Diego, CA, USA), GR-1 (Biolegend), F4/80 (Biolegend), TNF-*α* (R&D Systems), RBPMS (Phosphosolutions, Aurora, CO, USA), and CD31 (BD Biosciences, Mississuaga, ON, Canada) according to standard protocols. Following PBS-t washes, sections were incubated with fluorescent-conjugated 2° abs (Molecular Probes, Eugene, OR, USA) and mounted with DAPI. TUNEL staining was performed according to the manufacturer's instructions (DeadEnd; Promega, Fitchburg, WI, USA). Briefly, sections were fixed with 4% PFA for 5 min and washed in PBS. Equilibration buffer was added, and rTdT reaction mix was applied to each slide and incubated at 37 °C for 60 min. Slides were immersed in 2 × SSC and then washed with PBS, followed by blocking with 5% goat serum and incubation with 1° abs at 4 °C. Images were acquired with a Zeiss AxioImager fluorescence microscope. For *in vitro* staining, cells were washed, fixed in 4% PFA for 15 min, rinsed and permeabilized in 0.2% Triton X-100 for 15 min, and blocked with 5% BSA for 1 h. Cells were probed with 1° abs to: GFAP (Abcam), Vimentin (Sigma), or rhodamine phalloidin (Life Technologies, Burlington, ON, Canada), O/N at 4 °C, followed by washing and the appropriate Alexa Fluor 2° abs antibodies (Life Technologies) for 1 h. Cells were mounted with Vecta-Shield anti-fade medium with DAPI (Vector Labs, Burlingame, CA, USA), and imaged on an inverted Nikon TIE-E fluorescent microscope.

### Image analyses

To quantifying glial reactivity, retinal sections were imaged at the level of the optic nerve. The proportion of increased GFAP immunostaining in Müller cell processes was used as an established approach to quantify retinal glial reactivity.^43^ Briefly, for each animal GFAP-immunopositive processes were counted in the inner plexiform layer for at least five retinal sections at the level of the optic nerve. GFAP-positive processes were counted for each eye and expressed as the average number of positive processes per 100 *μ*m.^60^ To quantify the extent of apoptosis, we counted the number of TUNEL-positive nuclei in the GCL and expressed it as a fraction of the total GCL nuclei. In treated eyes the TUNEL signal was more difficult to find, so we conservatively counted any suspected labeling in our analyses. For each eye, at least five central retinal sections were analyzed at the level of the optic nerve stretching to each *ora serrata*, and the results averaged, as previously described.^28, 44, 45, 46, 83^ For TNF-*α* staining the mean intensity of antibody signal was measured in the GCL and normalized to the slide background for each section. Intensity readings were averaged for at least three sections at the level of the optic nerve for each eye, and then averaged across each treatment group as indicated. For all experiments eyes from at least three animals were assessed with specific numbers described in each figure legend. Statistical analyses were performed by one-way ANOVA with TUKEY *post hoc* analyses.

## Figures and Tables

**Table 1:** Media cytokines after WFA treatment

**Analyte**	**Normalized concentrations**	
	**Vehicle**	**2 *****μ*****M WFA**
TNF-*α*^a^	1.00	0.38
IL-1*α*	1.00	0.50
EGF	1.00	0.55
IL-1*β*	1.00	0.62
LIX	1.00	0.90
IL-6	1.00	0.91
RANTES	1.00	0.95
MIP-2	1.00	0.98
VEGF	1.00	1.01
IP-10	1.00	1.08
Leptin	1.00	1.16
IL-13	1.00	1.18
GRO/KC	1.00	1.20
IL-2	1.00	1.21
IL-12(p70)	1.00	1.22
IL-5	1.00	1.28
MCP-1	1.00	1.29
Fractalkine	1.00	1.45
IL-4	1.00	1.52
IFNy	1.00	1.59
IL-18	1.00	1.70
MIP-1*α*	1.00	1.74
GM-CSF	1.00	1.86
Eotaxin	1.00	2.24
IL-17A	1.00	2.39
IL-10	1.00	4.36
G-CSF	—	—

Abbreviation: WFA, withaferin A.

a *P*<0.05, *n*=3 isolates from eight animals each

## Abbreviations

GCL: ganglion cell layer
GFAP: glial fibrillary acidic protein
GS: glutamine synthetase
IF: intermediate filament
INL: inner nuclear layer
KA: kainic acid
MAPK: mitogen-activated protein kinase
SB: SB203580
RGC: retinal ganglion cell
WFA: withaferin A
