# Supplementary figures and images for: Pharmacologic inhibition of reactive gliosis blocks TNF-α-mediated neuronal apoptosis
Source: Cell Death Dis. 2016 Sep 29;7(9):e2386–. doi: 10.1038/cddis.2016.277 (PMC5059876; doi:10.1038/cddis.2016.277)

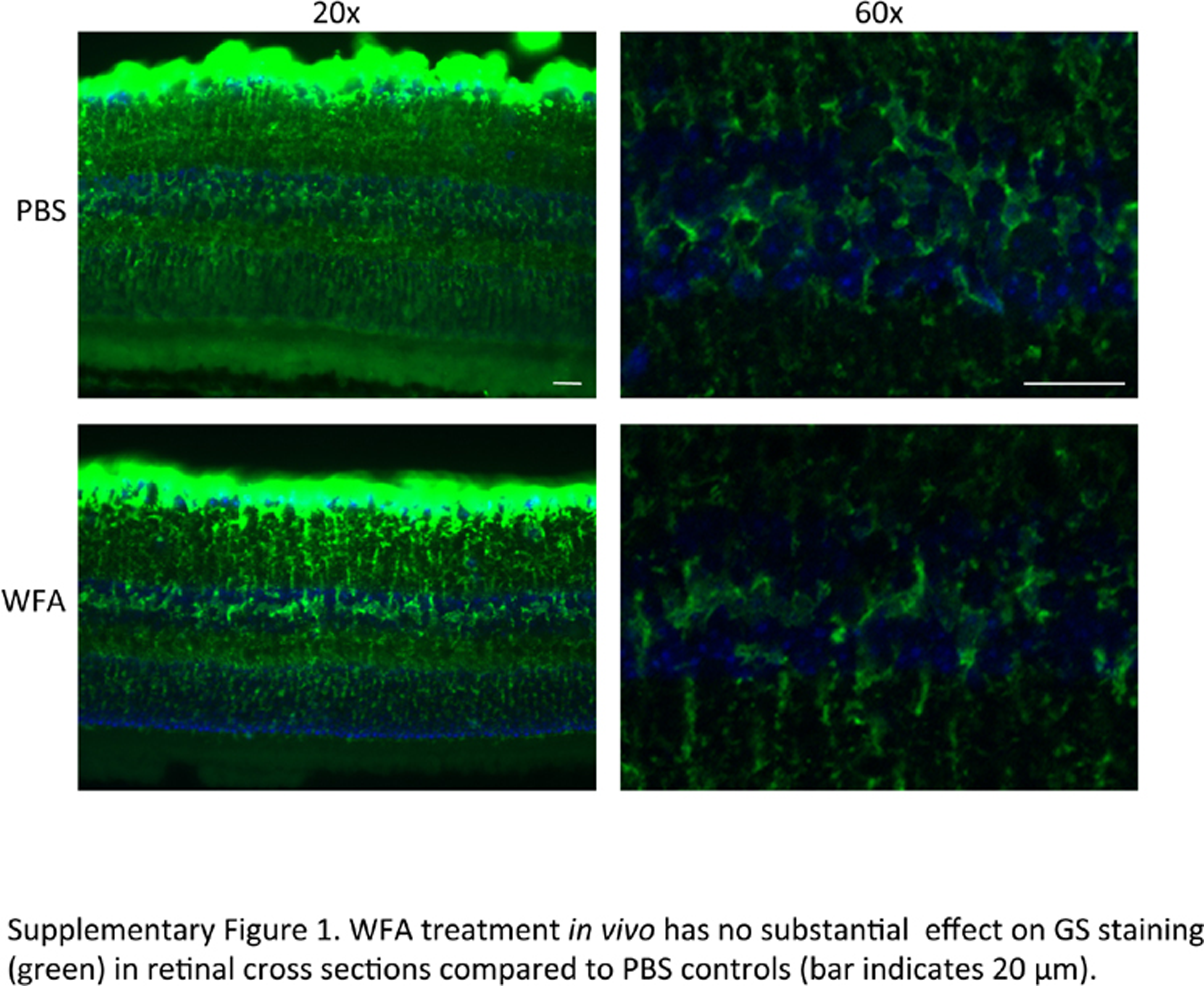

Supplement: Supplementary Figure 1 [file cddis2016277x1.tif]

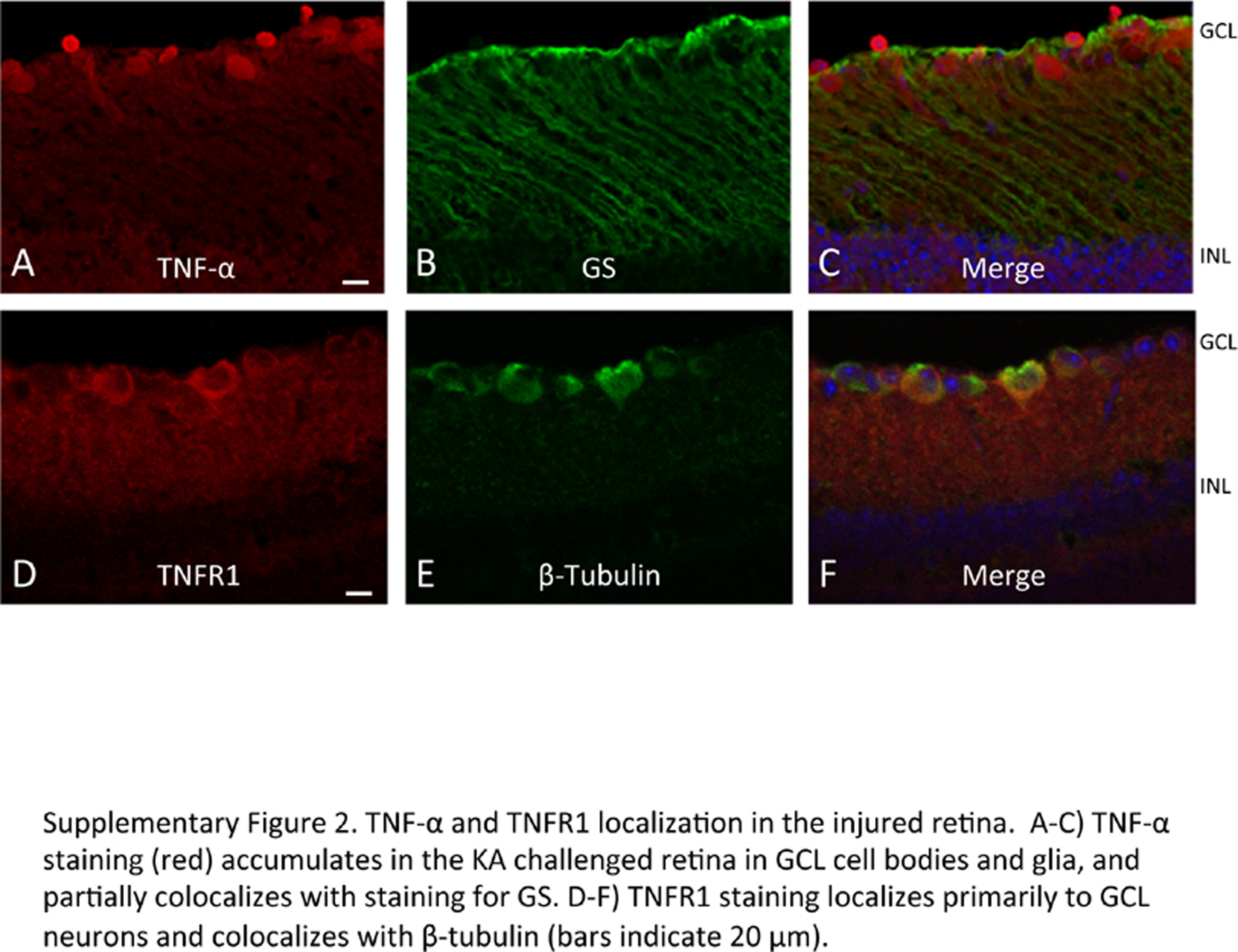

Supplement: Supplementary Figure 2 [file cddis2016277x2.tif]
